# Synthesis of Fluorine-Containing Analogues of Purine Deoxynucleosides: Optimization of Enzymatic Transglycosylation Conditions

**DOI:** 10.1134/S1607672922020053

**Published:** 2022-05-10

**Authors:** M. S. Drenichev, E. O. Dorinova, I. V. Varizhuk, V. E. Oslovsky, M. A. Varga, R. S. Esipov, D. D. Lykoshin, C. S. Alexeev

**Affiliations:** 1 Engelhardt Institute of Molecular Biology, Russian Academy of Sciences, Moscow, Russia; 2 Shemyakin–Ovchinnikov Institute of Bioorganic Chemistry, Russian Academy of Sciences, Moscow, Russia

**Keywords:** adenosine, 7-methylguanosine, nucleoside phosphorylase (NP), enzymes, transglycosylation, fluorine-containing derivatives, benzyladenine

## Abstract

In this work, a comparative analysis of the conditions of transglycosylation reactions catalyzed by *E. coli* nucleoside phosphorylases was carried out, and the optimal conditions for the formation of various nucleosides were determined. Under the optimized conditions of transglycosylation reaction, fluorine-containing derivatives of *N*^6^-benzyl-2'-deoxyadenosine, potential inhibitors of replication of enteroviruses in a cell, were obtained starting from the corresponding ribonucleosides.

## INTRODUCTION

**Fig 1.**
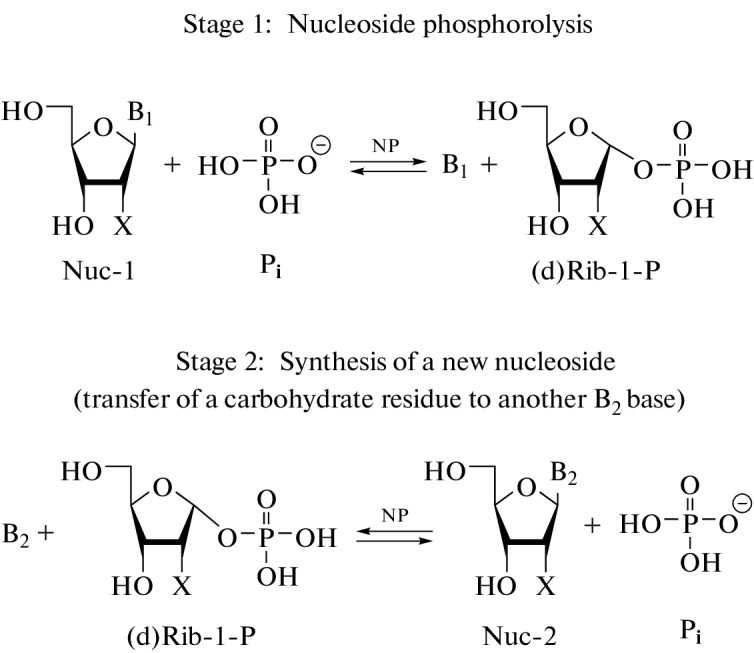
Enzymatic transglycosylation. Designations: NP, nucleoside phosphorylase; Nuc-1, nucleoside donor; Nuc-2, product; B1 and B2, heterocyclic bases; Pi, phosphate anion, X = H or OH. Reaction conditions: NP—*E. coli* PNP, *E. coli* UP, *E. coli* TP, 50 mM Tris-HCl (pH 7.5), 20°C. Compiled from [6].

Enzymatic transglycosylation methods are widely used for obtaining drugs on the basis of nucleosides and their analogues and are based on the reaction of transferring a carbohydrate residue from one heterocyclic base to another [1–3]. Nucleoside phosphorylases (NP), which perform reversible phosphorolysis of ribonucleosides/2'-deoxyribonucleosides with the formation of the corresponding heterocyclic base and α-*D*-(2-deoxy)ribofuranose-1-phosphate ((d)Rib-P). The equilibrium of the phosphorolysis reaction is shifted towards the formation of nucleosides, and in the case of purines it is more significant [1–6], which makes it possible to use two coupled reactions of phosphorolysis, a donor nucleoside and a nucleoside containing a heterocyclic base-acceptor, to perform an enzymatic transglycosylation reaction, during which a carbohydrate residue is transferred from a pyrimidine or purine nucleoside donor to a purine heterocyclic base acceptor (Fig. 1). This general scheme makes it possible to obtain new modified nucleosides depending on the set of used starting compounds and the substrate specificity of NP.

Earlier, approaches to optimizing the transglycosylation reaction using 7-methyl-2'-deoxyguanosine as the starting substrate for the production of α-*D*-2-deoxyribose-1-phosphate (dRib-1-P), 5-substituted derivatives of 2'-deoxyuridine, cladribine, and allopurinol-riboside [5, 7], were studied in the Laboratory of Design and Synthesis of Biologically Active Compounds (DSBAC) of the Engelhard Institute of Molecular Biology, Russian Academy of Sciences. A mathematical model of the transglycosylation process was proposed, which can be used to quantify the effect of initial conditions on the result of transglycosylation [8]. This work is a continuation of earlier studies aimed at expanding knowledge about the substrate specificity of NP and obtaining modified analogues of natural nucleosides by enzymatic transglycosylation.

## DISCUSSION

In this work, a comparative analysis of the transglycosylation reaction involving purine (PNP) and pyrimidine (UP, TP) *E. coli* nucleoside phosphorylases was performed (Table 1). The selection of enzymes of bacterial origin as catalysts was determined by their wide substrate specificity, pH optimum in neutral/weakly alkaline media, and a fairly wide operating temperature range, which allows the reaction to be carried out under mild conditions without noticeable nonspecific cleavage of the *N*-glycosidic bond with yields of target products close to the theoretically predicted ones [8–13].

**Table 1.** Comparative analysis of translycosylation conditions in the presence of *E. coli* nucleoside phosphorylases^1^

No.	Glycosyl-donor (D)	Base (B)	Product	D : B : P_i_ (mol)	Enzyme	Yield (HPLC), %	Conversion B to nucleoside (HPLC), %
1	Urd	Ade	Ado	1 : 1.5 : 1	UP/PNP	77	51
2	Urd	Ade	Ado	1.5 : 1 : 1	UP/PNP	88	88
3	Urd	Ade	Ado	3 : 1 : 2	UP/PNP	94	94
4	Ado	Ura	Urd	1 : 1.5 : 1	PNP/UP	22	13
5	Ado	Ura	Urd	1.5 : 1 : 1	PNP/UP	22	22
6	Ado	Ura	Urd	3 : 1 : 0.5	PNP/UP	27	27
7	Ado	Hyp	Ino	1 : 1.5 : 1	PNP	53	35
8	Ino	Ade	Ado	1 : 1.5 : 1	PNP	76	53
9	7-Me-Guo	Ura	Urd	1 : 1.5 : 1	PNP/UP	63	42
10	7-Me-Guo	Ura	Urd	1.5 : 1 : 1	PNP/UP	80	80
11	7-Me-Guo	Ade	Ado	1 : 1.5 : 1	PNP	79	52
12	7-Me-Guo	Ade	Ado	1.5 : 1 : 1	PNP	94	94
13	7-Me-Guo	Ade	Ado	3 : 1 : 2	PNP	100	99
14	Rib-P	Ade	Ado	1 : 1.5	PNP	98	66
15	Thd	5-Et-Ura	5-Et-Urd	5 : 1 : 0.5	TP	80	80
16	7-Me-dGuo	PFPh-Ade	PFPh-dAdo	1.5 : 1 : 1	PNP	89	89
				1.5 : 1 : 0.5		95	95
				1.5 : 1 : 0.25		97	97
17	7-Me-dGuo	TFMBn-Ade	TFMBn-dAdo	1.5 : 1 : 1	PNP	96	96
				1.5 : 1 : 0.5		98	98
				1.5 : 1 : 0.25		100	100

^1^Reaction conditions: The reactions were performed in 1.5-mL plastic tubes (Eppendorf) in 1 mL of 50 mM Tris-HCl (pH 7.5), D : B : Pi concentrations 0.01–1 mM, 0.35 units of *E. coli* PNP, 0.34 units of *E. coli* UP.

^2^Analysis conditions: linear gradient 2–12% CH_3_CN in 0.08% aq. TFA for 10 min, 4.6 × 150 mm Luna C18(2) column (lines 1–6, 9–15); 0.5–3% CH_3_CN in 0,08% aq. TFA for 15 min, 4.6 × 150 mm Luna C18(2) column (lines 7, 8); 2–60% CH_3_CN in 10 mM aq. sodium acetate for 25 min, 4.6 × 250 mm Nucleosil C18 (lines 16, 17).

According to Fig. 1, the transglycosylation reaction proceeds through the formation of α-*D*-ribose-1-phosphate (Rib-1-P) or α-*D*-2-deoxyribose-1-phosphate (dRib-1-P). The synthesis of purine nucleosides from pyrimidine nucleosides and vice versa requires the participation of two enzymes: PNP and UP (or TP). The equilibrium constants of phosphorolysis of natural pyrimidine nucleosides in the presence of UP and TP are higher than the equilibrium constants of phosphorolysis of natural purine nucleosides in the presence of PNP [2, 5, 8, 11, 14]. Therefore, it is more reasonable to use Rib-1-P and dRib-1-P (or pyrimidine, but not purine, nucleosides) as donors [8, 15, 16].

This expediency is well confirmed by the experimental data presented in Table 1.

When adenosine was obtained from uridine, the reaction proceeded with a high yield, which was determined as the ratio of the equilibrium concentration of the product to the initial concentration of the starting base or glycosyl donor, depending on what was taken in deficiency (Table 1, lines 1–3, 77–94% according to HPLC). When uridine was obtained from adenosine, the reaction yield was significantly reduced (Table 1, lines 4–6, 22–27% according to HPLC). In the series of purine nucleosides, inosine was a more productive glycosyl donor than adenosine; therefore, the reaction for obtaining adenosine from inosine proceeded with a higher yield (Table 1, line 8, 76% according to HPLC data) than the reverse reaction (Table 1, line 7, 53% by HPLC). With an increase in the amount of the donor nucleoside in the reaction mixture, it is naturally possible to increase the yield of the target nucleoside (and, accordingly, the conversion of the base); however, this also increases the number of unreacted components, which increases the complexity of the subsequent processing of the reaction mixture and further purification (Table 1, lines 3, 6, 13, 15).

The transglycosylation reaction can be simplified in two ways:

(1) exclusion of the stage of phosphorolysis of the nucleoside that serves as a glycosyl donor in the transglycosylation reaction (Fig. 1, stage 1) by introducing a ready-to-use Rib-1-P or dRib-1-P into the reaction [7];

(2) transformation of stage 1 into an irreversible one using 7-methyl-(2'-deoxy)guanosine (7-Me(d)Guo) as a source of ribose residue due to its almost irreversible phosphorolysis [17, 18].

The use of (d)Rib-1-P reduces the number of components in the reaction mixture, facilitates the isolation of target compounds, and makes it possible to significantly shift the equilibrium of the glycosylation reaction towards the formation of nucleosides (Table 1, line 14, 98% according to HPLC; the yield was calculated from the reagent taken in deficiency (Rib-1-P)). The replacement of (d)Rib-1-P with the more accessible 7-Me(d)Guo leads to comparable yields of the reaction products (Table 1).

**Fig. 2.**
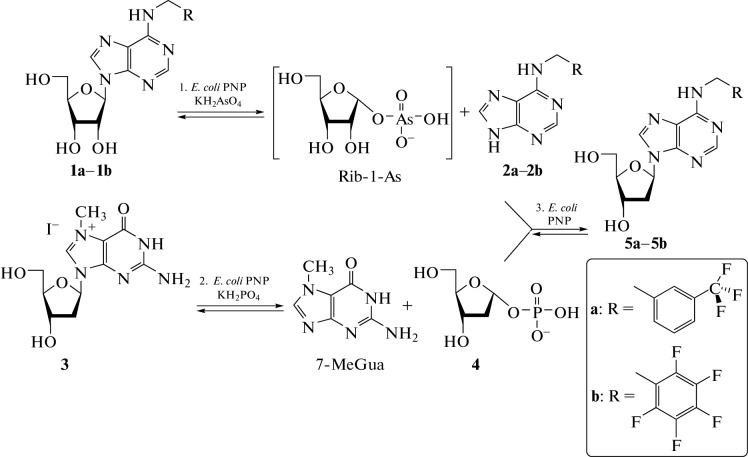
Synthesis of fluoro substituted 2'-deoxyribonucleosides from ribonucleosides. Reagents and conditions: (1) **1a–1b** (0.212–0.235 mmol), *E. coli* PNP (0.25 units), KH_2_AsO_4_ (0.212–0.235 mmol), 50 mM Tris-HCl buffer (pH 7.5, 10 mL), 50°C, 20 h—**2a** (91%), **2b** (85%). (2–3) **2a** (20 mg, 0.068 mmol), **2b** (20 mg, 0.064 mmol), **3** (0.102 mmol), *E. coli* PNP (0.98 U), КH_2_PO_4_ (0.064–0.068 mmol), 50 mM Tris-HCl (pH 7.5, 63 mL)—DMSO (10%, 7 mL), 20°C, 24 h—86% (**5a**), 47% (**5b**).

*E. coli* PNP was used to prepare less available deoxyribonucleosides from commercially available ribonucleosides, *N*^6^-pentafluorophenylmethyl-2'-deoxyadenosine (PFPh-dAdo, **5b**) and *N*^6^-(3-trifluoromethylbenzyl)-2'-deoxyadenosine (TFMBn-dAdo, **5a**) (Fig. 2), potential inhibitors of enterovirus replication in the cell [19].

The synthesis method consisted of three separate stages, each stage was catalyzed by *E. coli* PNP. The starting bases *N*^6^-(3-trifluoromethylbenzyl)adenine (TFMBn-Ade, **2a**) and *N*^6^-pentafluorophenylmethyladenine (PFPh-Ade, **2b**) were obtained from ribonucleosides under the conditions of enzymatic arsenolysis (Fig. 2, stage 1). Enzymatic arsenolysis is based on the cleavage of ribonucleoside in the presence of potassium dihydroorthoarsenate (KH_2_AsO_4_) into a purine base and the highly labile α-*D*-ribofuranose-1-arsenate (Rib-1-As), which is irreversibly hydrolyzed; this shifts the equilibrium of ribonucleoside cleavage towards the formation of a base [20]. The poor solubility of heterocyclic bases **2a** and **2b** in water and in Tris-HCl buffer also leads to a shift in equilibrium towards the formation of products. To prevent the formation of a mixture of ribo- and deoxyribonucleosides during further stages, stage 1 should be performed in a separate flask. Then, bases **2a** and **2b** were filtered off and introduced into the transglycosylation reaction with 7-Me-dGuo in the presence of potassium dihydroorthophosphate and *E. coli* PNP (Fig. 2, stages 2–3). In the reaction mixture, 7-Me-dGuo was converted into dRib-1-P (Fig. 2, stage 2), which then reacted with a fluorine-containing base (Fig. 2, stage 3). Stages 2 and 3 were performed in the same flask. To increase the solubility of the base, the reaction was carried out in a buffer solution with the addition of 10 vol % dimethyl sulfoxide. 

The concentration of dimethyl sulfoxide in the reaction mixture did not significantly affect the enzymatic activity of PNP, which is consistent with the literature data [18]. The transglycosylation reaction was carried out at different glycosyl donor : base : phosphate ratios (Table 1). Carrying out the reaction with a slight excess of the glycosyl donor in the presence of an equimolar amount of phosphate (1.5 : 1 : 1) or its deficiency (1.5 : 1 : 0.5, 1.5 : 1 : 0.25) led to high yields of target nucleoside products with a slight decrease in the reaction rate compared to the reaction rate in the presence of equimolar amounts of phosphate. An increase in the amount of phosphate in the reaction mixture (starting from an equimolar amount and above) leads to an increase in the rate of formation of dRib-1-P, the hydrolysis of which can reduce the yields of target nucleosides (Table 1, lines 16–17). Therefore, the highest yields were achieved at a glycosyl donor : base : phosphate ratio of 1.5 : 1 : 0.25 (Table 2). The yield of the preparative method for obtaining the **5a** product was 100% by HPLC (86% after purification by reverse-phase chromatography on silica gel-C18), and the yield of the **5b** product was 92% by HPLC (47% after similar purification, the low yield was due to the sorption of the compound on silica gel-C_18_).

**Table 2.** HPLC-analysis of compound **5a** formation from base **2а** and 7Me-dGuo (**3**) in the presence of *E. coli* PNP


**A** (*t*_initial_): (*1*) 7Me-dGuo (**3**); (*2*) TFMBn-Ade (**2a**)

**B** (35 min): (*2*) 7Me-dGuo (**3**); (*3*) 7-MeGua; (*4*) TFMBn-dAdo (**5a**), (*5*) TFMBn-Ade (**2a**)

**C** (*t*_equilibrium_): (*1*) 7-MeGua, (*2*) TFMBn-dAdo (**5a**)

Analysis conditions: Nucleosil 100–5 C18 4.6 × 250 мм (5 µm, Macherey–Nagel GmbH&Co. KG), MeCN in 10 mM NaOAc/H_2_O from 2 to 60% (25 min) (with further flushing with 60–80% MeCN/10 mM NaOAc/H_2_O for 25–25.1 min, then 80–2% for 25.1–25.9 min), flow 1 mL/min, UV 265 nm

The structure of the obtained compounds was confirmed by NMR spectroscopy. The ^13^С-NMR spectrum of the trifluoromethyl-substituted deoxynucleoside **5а** contained a resonance signal of the trifluoromethyl group in the form of a low-intensity quartet with a spin–spin coupling constant (SSCC) ^1^*J*_C–F_ = 31 Hz. The ^13^С-NMR spectrum of the pentafluoro-substituted deoxynucleoside **5b** contained resonance signals of the ^13^С nuclei of the phenyl group in the form of three wide doublets with SSCC ^1^*J*_C–F_ of approximately 250 Hz. In the ^19^F-NMR spectrum, the signal of the trifluoromethyl group of compound **5а** was resolved as a singlet with a chemical shift δ = 61.04 ppm. The ^19^F-NMR spectrum of the pentafluoro-substituted nucleoside **5b** shows a complex spin–spin interaction: a doublet of doublets for the ^19^F nuclei in the *ortho* position of the phenyl substituent with SSCC ^3^*J*_F–F_ = 22 Hz, ^4^*J*_F–F_ = 6 Hz, a triplet of doublets for the ^19^F nuclei in the *meta* position with SSCC ^3^*J*_F–F_ = 22 Hz, ^4^*J*_F–F_ = 6 Hz and a triplet for the ^19^F nuclei in the *para* position of the phenyl ring with SSCC ^3^*J*_F–F_ = 22 Hz. The presence of a pentafluoro-substituted fragment in the **5b** structure was also confirmed by the ^1^H-NMR spectrum, in which no resonance signals of the phenyl group protons were observed.

The antiviral activity of the obtained compounds is currently being studied.

## EXPERIMENTAL

Commercially available reagents and solvents were used to carry out reactions and isolate compounds. The course of reactions was monitored by HPLC analysis on a Styer-M instrument (Akvilon, Russia). HPLC analysis conditions: column 4.6 × 250 mm (Nucleosil 100-5 C18, 5 µm, Macherey-Nagel GmbH&Co. KG), linear gradient of acetonitrile in 10 mM sodium acetate solution in deionized water from 2 to 60% over 25 min (with further washing in the system 60–80% acetonitrile/10 mM NaOAc in H_2_O for 25–25.1 min and then 80–2% for 25.1–25.9 min) at a flow rate of 1 mL/min. UV detection was performed at 265 nm, sample volume was 20 µL. NMR spectra were recorded on a Bruker AMX 400 and Bruker AMX 300 instruments (Germany). The values of the spin–spin coupling constants (SSCC, *J*) are measured in hertz (Hz). When describing NMR spectra, the following abbreviations are used: s—singlet, br s—broadened singlet, d—doublet, dd—doublet of doublets, ddd—doublet of doublets of doublets, t—triplet, dt—doublet of triplets, m—multiplet. The ^1^Н- and ^13^С-NMR spectra were calibrated using the residual signal of the solvent DMSO-*d*_6_ (2.50 and 39.52 ppm, respectively).

### Preparation of N^6^-(2,3,4,5,6-pentafluorobenzyl)adenine (**2b**)

A mixture of *N*^6^-(2,3,4,5,6-pentafluorobenzyl)adenosine **1b** (95 mg, 0.212 mmol) and KH_2_AsO_4_ (38 mg, 0.212 mmol, 1 equiv.) in 50 mM Tris-HCl buffer (pH 7.5, 10 mL) was supplemented with PNP (0.050 mL, 5 activity units), and the mixture was incubated at 50°C for 20 h. During the reaction, the initial nucleoside was dissolved and the product was crystallized. After 20 h of incubation, the reaction mixture was cooled to room temperature and incubated for 24 h at 4°C. The formed precipitate was filtered off, washed with water (5 × 5 mL), and dried in a vacuum desiccator over P_2_O_5_ for 1 day. Yield 57 mg (85%) as white powder. R_*f*_ = 0.4 (CH_2_Cl_2_ : EtOH-95 : 5 ${\text{v/v}}$). ^1^H–NMR (300 МHz, DMSO-*d*_6_): δ = 12.96 (br s, 1H, N9H), 8.21 (s, 1H, H2-Ade), 8.15 (br s, 1H, *N*^6^H), 8.11 (s, 1H, H8-Ade), 4.83 (br s, 1H, *N*^6^CH_2_). ^19^F-NMR (282 МHz, DМSO-*d*_6_): δ = –142.50 (dd, ^3^*J*_F–F_ = 24.0 Hz, ^4^*J*_F–F_ = 7.9 Hz), –156.85 (t, ^3^*J*_C–F_ = 22.1), –163.68 (td, ^3^*J*_F–F_ = 23.1 Hz, ^4^*J*_F–F_ = 7.8 Hz).

### Preparation of N^6^-(3-trifluoromethylbenzyl)adenine (**2a**)

The procedure is similar to the preparation of *N*^6^-(2,3,4,5,6-pentafluorobenzyl)adenine (**2b**) from *N*^6^-(3-trifluoromethylbenzyl)adenosine (**1a**) (100 mg, 0.235 mmol). Yield **2a** 63 mg (91%) as white powder. R_*f*_ = 0.42 (CH_2_Cl_2_ : EtOH – 95 : 5 ${\text{v/v}}$). ^1^H–NMR (300 МHz, DMSO-*d*_6_): δ = 12.95 (br s, 1H, N9H), 8.29 (br s, 1H, *N*^6^H), 8.17 (s, 1H, H2–Ade), 8.12 (s, 1H, H8-Ade), 8.0–7.4 (м, 4H, Ph), 4.81 (br s, 1H, *N*^6^CH_2_). ^19^F-NMR (282 МHz, DМSO-*d*_6_): δ = –61.00.

### Preparation of N^6^-(3-trifluoromethylbenzyl)-2'-deoxyadenosine (**5a**)

Solution (70 mL) containing 7-methyl-2'-deoxyguanosine (41.82 mg, 0.102 mmol), meta-trifluoromethylbenzyladenine **2a** (20.00 mg, 0.068 mmol), and potassium dihydrogen phosphate (2.32 mg, 0.017 mmol) in 50 mM Tris-HCl buffer (pH 7.5) with the addition of 10 vol % DMSO was supplemented with 0.98 units of *E. coli* PNP (10 µL of 1.0 mg/mL Sigma solution at a concentration of 98 units/mL) at room temperature. The mixture was gently stirred for 5 min and left at room temperature for 24 h. The precipitate of 7-methylguanine was filtered off through a Phenomenex nylon membrane (diameter 47 mm, pore size 0.2 µm). The filtered clear solution was evaporated in vacuo to a volume of ~7 mL, diluted with water (7 mL), and applied onto a column with a reverse-phase sorbent C18. Yield: 99% (HPLC, quantitative). The column was washed with a water : ethanol mixture (ethanol concentration gradient of 0–20%). The product was eluted in the water : ethanol mixture 60 : 40. The fractions containing the product were pooled, evaporated in vacuo, and coevaporated with ethanol. Yield after isolation and purification was 26 mg (86%) as a foam. ^1^H-NMR (400 МHz, DMSO-*d*_6_): δ = 8.51 (br s, 1H, *N*^6^H-Ade), 8.38 (s, 1H, H2-Ade), 8.21 (s, 1H, H8-Ade), 7.71 s (1H, *о-*Н, Ph), 7.65 d (1Н, ^3^*J* = 7.2, *p*-H, Ph), 7.60–7.49 m (2H, Ph), 6.36 dd (1H, *J*_1'2'a_= 7.6, *J*_1'2'b_ = 6.2, H-1'), 5.32 br s (1H, 3'-OH), 5.19 br s (1H, 5'-OH), 4.79 br s (2H, CH_2_), 4.46–4.38 m (1H, H-3'), 3.89 td (1Н, *J*_4'5'а_ = *J*_4'3'_ = 4.2, *J*_4'5'b_ = 2.6, H-4'), 3.62 br d (1Н, *J*_5'а5'b_ = –11.7, H-5'a), 3.52 br d (1Н, *J*_5'b5'a_ = –11.7, H-5'b), 2.73 ddd (1Н, *J*_2'а2'b_= –13.3, *J*_2'а1'_= 7.6, *J*_2'b3'_ = 5.9, H-2'а), 2.27 ddd (1Н, *J*_2'b2'a_= –13.3, *J*_2'b1'_= 6.2, *J*_2'b3'_ = 2.6, H-2'b). ^13^C-NMR (150 МHz, DMSO-*d*_*6*_): 154.37 (C-2), 152.32 (C-4), 141.59 (C-6), 139.74 (C-8), 131.33 (Ph), 129.29 (Ph), 128.90 q (^1^*J*_C–F_ = 31.4, CF_3_), 123.65 q (^3^*J*_C–F_ = 3.8, Ph), 123.43 q (^3^*J*_C–F_ = 3.7, Ph), 119.68 (C-5), 88.03 (C-1'), 83.97 (C-3'), 70.94 (C-4'), 61.87 (C-5'), 42.62 (CH_2_), 39.47 (C-2'). ^19^F-NMR (282 МHz, DМSO-*d*_6_): δ = –61.04.

### Preparation of N^6^-(2,3,4,5,6-pentafluorophenyl-1-methyl)-2'-deoxyadenosine (**5b**)

The procedure is similar to the previous one, starting from *N*^*6*^-(2,3,4,5,6-pentafluorobenzyl)adenine **2b** (20.00 mg, 0.064 mmol). Purification was carried out on a column with a reversed-phase sorbent C_18_ using a water : ethanol mixture (ethanol concentration gradient of 0–20%) as a mobile phase. Yield 13 mg (47%) as white silver foam. ^1^H-NMR (400 МHz, DMSO-*d*_6_): δ = 8.37 (s, 1H, H2-Ade), 8.35 (br s, 1H, *N*^6^H-Ade), 8.25 (s, 1H, H8-Ade), 6.35 dd (1H, *J*_1'2'a_= 7.6, *J*_1'2'b_ = 6.2, H-1'), 5.30 br s (1H, 3'-OH), 5.14 br t (1H, *J*_5'OH_ = 4.2, 5'-OH), 4.82 br s (2H, CH_2_), 4.45–4.37 m (1H, H-3'), 3.87 td (1Н, *J*_4'5'а_ = *J*_4'3'_ = 4.3, *J*_4'5'b_ = 2.8, H-4'), 3.62 br d (1Н, *J*_5'а5'b_ = –11.7, H-5'a), 3.55–3.45 m (1Н, H-5'b), 2.72 ddd (1Н, *J*_2'а2'b_= –13.3, *J*_2'а1'_= 7.7, *J*_2'b3'_ = 5.8, H-2'а), 2.27 ddd (1Н, *J*_2'b2'a_= –13.3, *J*_2'b1'_= 6.1, *J*_2'b3'_ = 2.8, H-2'b). ^13^C-NMR (150 МHz, DMSO-*d*_6_): 153.85 (C-2), 152.14 (C-4), 148.57 (C-6), 145.10 dddd (^1^*J*_C–F_ = 245.8, ^2^*J*_C–F_ = 13.2, ^2^*J*_C–F_ = 9.1, ^3^*J*_C–F_ = 3.9, *ortho*-С_6_F_5_), 139.79 (C-8), 139.68 dddd (^1^*J*_C–F_ = 250.5, ^2^*J*_C–F_ = 13.7, ^2^*J*_C–F_ = 12.4, ^3^*J*_C–F_ = 5.6, *meta*-С_6_F_5_), 136.69 dtt (^1^*J*_C–F_ = 248.8, ^2^*J*_C–F_ = 12.6, ^3^*J*_C–F_ = 3.9, *para*-С_6_F_5_), 119.65 (C-5), 113.01 t (^2^*J*_C–F_ = 17.6, *C-1*-C_6_F_5_), 87.98 (C-1'), 83.88 (C-3'), 70.88 (C-4'), 61.81 (C-5'), 39.42 (C-2'), 32.22 (CH_2_). ^19^F-NMR (282 МHz, DМSO-*d*_6_): δ = –142.50 dd (^3^*J*_F–F_ = 22, ^4^*J*_F–F_ = 6), –156.74 t (^3^*J*_F–F_ = 22), –163.65 td (^3^*J*_F–F_ = 22, ^4^*J*_F–F_ = 6).

## CONCLUSIONS

In the study, a comparative analysis of the conditions of transglycosylation in the presence of *E. coli* nucleoside phosphorylases was carried out, which made it possible to optimize the conditions for the enzymatic synthesis of *N*^6^-pentafluorophenylmethyl-2'-deoxyadenosine and *N*^6^-(3-trifluoromethylbenzyl)-2'-deoxyadenosine, potential nucleoside inhibitors of enterovirus replication in cells.
